# Acute hepatitis C virus infection assessment among chronic hemodialysis patients in the Southwest Parana State, Brazil

**DOI:** 10.1186/1471-2458-7-50

**Published:** 2007-04-04

**Authors:** Maricea Engel, Fernanda M Malta, Michele MS Gomes, Isabel MVGC Mello, João RR Pinho, Suzane K Ono-Nita, Flair J Carrilho

**Affiliations:** 1 Department of Gastroenterology, Hepatology Branch, University of São Paulo, School of Medicine, São Paulo, Brazil

## Abstract

**Background:**

Chronic hemodialysis patients are at higher risk for acquiring hepatitis C virus (HCV). The prevalence varies among different countries and hemodialysis centers. Although guidelines for a comprehensive infection control program exist, the nosocomial transmission still accounts for the new cases of infection. The aim of this study was analyze the follow up of newly acquired acute hepatitis C cases, during the period from January 2002 to May 2005, in the Hemodialysis Center, located in the Southwest region of Parana State, Brazil and to analyze the effectiveness of the measures to restrain the appearance of new cases of acute hepatitis C.

**Methods:**

Patients were analyzed monthly with anti-HCV tests and ALT measurements. Patients with ALT elevations were monitored for possible acute hepatitis C.

**Results:**

During this period, 32 new cases were identified with acute hepatitis C virus infection. Blood screening showed variable ALT levels preceding the anti-HCV seroconversion. HCV RNA viremia by PCR analysis was intermittently and even negative in some cases. Ten out of 32 patients received 1 mcg/kg dose of pegylated interferon alfa-2b treatment for 24 weeks. All dialysis personnel were re-trained to strictly follow the regulations and recommendations regarding infection control, proper methods to clean and disinfect equipment were reviewed and HCV-positive patients were isolated.

**Conclusion:**

Laboratory tests results showed variable ALT preceding anti-HCV seroconversion and intermittent viremia. The applied recommendations contributed importantly to restrain the appearance of new cases of acute hepatitis C in this center and the last case was diagnosed in May 2004.

## Background

Hepatitis C virus (HCV) prevalence differs among hemodialysis units according to their geographical location, health care procedures, socioeconomic factors, reuse of lines, hygiene and sterilization of equipment, patient rotation of machines and the undertaking of rigorous universal precaution rules. These features influence the risk of nosocomial transmission of HCV to hemodialysis patients [1-3]. Previous reports reinforces the importance of vigilance in these units [2,4,5], especially the strict following of recommendations to prevent the transmission of infectious diseases among these patients [6].

In chronic renal patients, hepatitis C virus infection is often difficult to evaluate. The results of assays used to diagnose this infection can be hard to interpret due to the particular features found in this population. The identification of acute HCV infection cases can be influenced by several factors: a) absence of symptoms in acute cases, the majority of them without jaundice and with symptoms related to the base of the disease or to chronic anemia; b) slight increases of aminotransferases levels; C) anti-HCV negative serology or positive after several months of contamination; and d) intermittent viremia. Patients presenting intermittent viremia were described in 33% to 67% of anti-HCV positive patients [7-9]. The levels of alanins aminotransferase (ALT) are generally lower in patients with renal failure. Previous reports shown the need to establish lower ALT levels standard to increase the accuracy of this assay in detecting liver diseases [10-12].

The aims of this study were to follow the development of acute hepatitis C cases in a renal therapy unit located in the southeast of Parana from January 2002 to May 2005, and the response to the measures taken to avoid new acute hepatitis C cases.

## Methods

### Study design

From January 2002 to May 2005, we prospectively followed up all patients at a single hemodialysis unit in Pato Branco city, southeast Parana, Brazil. The study patients were prospectively tested every month by a third-generation anti-HCV ELISA. Positive results obtained by ELISA test were confirmed by HCV RNA testing. ALT determination in the study group was performed each month.

### Determination of HCV RNA and HCV genotype

Serum HCV RNA was detected as follows: RNA was extracted from 100 micro liters of serum by guanidinium isothiocyanate, phenol chloroform extraction described by Chomczynski & Sacchi [13]. Complementary DNA was synthesized using random primers and reverse transcriptase Moloney Murine Leukemia Virus (M-MLV) (Invitrogen Life Technologies, Carlsbad, CA, USA). HCV RNA was detected by two-stage polymerase chain reaction (PCR) using primers from the 5' – untranslated region of the HCV genome, as previously described [14]. HCV genotypes of the patients with HCV viremia were determined by sequencing (Applied Biosystems, Foster city, CA, USA), as previously described [14].

### IFN therapy and follow-up

Infected patients were informed of the possible risks and benefits of IFN treatment. Ten out 32 patients diagnosed with acute hepatitis C were treated with pegylated interferon 1 mcg/kg/weekly for 24 weeks. Pegylated IFN-a 2b (Intron A; Schering-Plough Corp., Kenilworth, NJ) was patient-administered subcutaneously at doses of 1 microgram/kg/weekly for 24 weeks. Each patient check-up included measurement of serum ALT levels and a complete blood count, and this was done in the outpatient clinic at 1-week intervals during the first month, at 2-week intervals during the second and third months of treatment, and monthly after the end of the treatment. Serum HCV RNA was assessed qualitatively before treatment, at end of treatment and at month 6 post-treatment. Sustained virological response was considered in cases with undetectable HCV RNA 6 months after the end of treatment.

All patients gave written informed consent. The study was approved by the local hospital ethical committee and Clinical Hospital of the University of São Paulo School of Medicine.

### Infection control training and education

After the detection of new acute hepatitis C cases, more stringent precautions were taken at the hemodialysis units to reduce the appearance of new cases.

The measures were based on different publications and recommendations [3,6,15-17] and in brief are summarized as follows:

a) Reeducation of existing staff members regarding Recommended Infection Control Practices for Hemodialysis.

b) All the equipment used on patients with positive serology, such as stethoscope, sphygmomanometer, and Micropore dressings were separated. Isolated carts were installed for use according to anti-HCV serology.

c) Patients attending the unit who had been transferred from another centre and with ALT alterations were isolated until serological confirmation.

d) As in 2002 new cases continued to arise, it was decided to separate patients with anti-HCV positive to undergo dialysis sessions on Mondays, Wednesdays and Fridays, while the anti-HCV negative patients to Tuesdays, Thursdays and Saturdays, always utilizing the same machine for each patient. The transportation of patients was separated in accordance to HCV serology or HCV-RNA because of the possibility of episodes of vascular bleeding (fistula) due to heparinization.

e) Hemodialysis machines were sterilized after each session of dialysis with Persteril 3.5 [peracetic acid 3.5%] and externally cleaned with soap and water. There were also changes in the sterilizing procedures of the lines (reuse). The lines were sterilized in separate rooms, conforming to serology (anti-HCV positive, anti-HCV negative HBsAg positive), in semi-automatic reprocessing device for up to 10 lines, to sterilizing the lines of patients with anti-HCV negative serology in individual automatic reprocessing device (Renatron, Minntech Renal Systems) with hydrogen peroxide 20%, peracetic acid 4.5%. The lines were reused in accordance with the internal number of them (priming), being discarded with priming < 80% or depending on its reuse, no more than 12 times when reprocessed manually, or up to 20 times when reprocessed automatically, in agreement with the resolution – RDC # 154, 15^th ^of July 2004 [18].

f) The health-care workers from the unit were examined for anti-HCV (12 nursing technicians, 2 nurses, 2 nephrology doctors and 2 maintenance workers) and were all serologically negative.

## Results

Table 1 shows the number of patients followed at the hemodialysis unit. We identified by anti-HCV ELISA 32 patients with acute HCV infection over the study period. A higher number of cases at the initial stages of the studies were diagnosed and their number decreased over the subsequent years (Table 2).

**Table 1:** Number of patients followed at the hemodialysis center and acute hepatitis C new cases

	2002	2003	2004	May/2005
Initial total number of patients	74	86	87	71
Admissions	40	32	37	21
Deaths	12	15	18	04
Drop-outs	13	09	16	09
Kidney transplantations	03	07	19	03
Total number of patients	86	87	71	76
Total acute hepatits C new cases	20	06	06	00

**Table 2:** ALT levels 4 months before anti-HCV seroconversion from patients followed up from January 2002 to May 2005

Case number	ALT levels 4 months before seroconversion				ALT/Anti-HCV seroconversion month
01	17	12	28	223	20/March 2002
02	10	28	325	253	179/March 2002
03	27	162	248	13	19/April 2002
04	16	21	76	250	15/May 2002
05	26	34	26	63	280/May 2002
06	12	11	57	179	56/May 2002
07	14	13	17	22	19/May 2002
08	24	19	34	33	63/May 2002
09	22	52	73	164	178/May 2002
10	18	35	85	270	26/May 2002
11	03	25	18	78	179/July 2002
12	25	14	143	111	203/July 2002
13	04	05	15	250	318/July 2002
14	15	26	13	226	31/August 2002
15	11	04	25	253	187/August 2002
16	61	69	201	61	190/September 2002
17	07	22	29	246	159/September 2002
18	16	29	84	102	73/September 2002
19	66	17	18	45	124/September 2002
20	25	37	30	105	275/November 2002
21	26	39	183	100	304/January 2003
22	05	07	38	174	110/January 2003
23	04	14	50	111	18/January 2003
24	37	40	39	28	114/January 2003
25	14	69	318	158	99/November 2003
26	11	38	31	85	52/November 2003
27	08	24	186	36	08/January 2004
28	12	51	114	195	223/January 2004
29	14	08	12	94	-/February 2004
30	26	28	41	33	203/March 2004
31	-	-	14	24	103/April 2004
32	07	11	63	54	54/May 2004

Ten patients were selected for treatment. We used pegylated interferon alfa 2b, at dose of 1 mcg/Kg body weight, during 24 weeks. The selected patients could not be standardized in relation to the onset of the disease. Patients started treatment an average 6.9 ± 3.5 months after the first HCV was positive. Of the 10 patients treated, four (cases 14, 16, 23, 32) had sustained virological response, three (cases 15, 19, 24) did not responded to treatment, one patient (case 31) died during treatment, owing to fistula infection and pneumonia, without evidence of leukopenia. One patient (case 27) with a high ALT level and anti-HCV seroconversion received treatment and was HCV-RNA negative in the samples collected thereafter. Another patient (case 25), from whom samples of HCV-RNA were taken 3 months prior to treatment, did not showed a positive PCR, therefore we could not determine if these two cases responses were due to natural causes or therapeutic response.

At the end of the investigation, among the 32 cases with acute HCV infection, 12 patients had detectable PCR-RNA (one case treated), 10 had PCR-RNA not detected (six treated), nine deaths (three treated) and the loss of one case report (hemodialysis unit changed)

HCVgenotyping showed 22 cases infected with type 1 three cases with type 3 (Table 3). In seven cases, it was not possible to carry out the genotyping because of negative HCV RNA at the samples.

**Table 3:** HCV genotype from the 32 cases of acute HCV

Genotype	Total
1	22
3	3
Not done	7
Total	32

Liver biopsy was carried out on just three patients, without adverse events, all presenting slight histological alterations according to the Brazilian Society of Pathology classification. A histological study was not carried out on the other patients. After the detection of new acute hepatitis C virus cases and their treatment, measures were taken to restrain other detected outbreaks and reduce the appearance of new cases.

The measures established were based on different publications and recommendations by the National Agency of Sanitary Vigilance [18,3,6,15-17] and followed as described above in the Material and Methods section.

As in 2002 new cases continued to arise, it was decided to separate patients with anti-HCV positive to undergo dialysis sessions on Mondays, Wednesdays and Fridays, while the anti-HCV negative patients to Tuesdays, Thursdays and Saturdays, always utilizing the same machine for each patient.

With the measures established the hepatitis C outbreak was controlled and last case of acute hepatitis C seen in this unit was May 2004.

## Discussion

Nosocomial transmission of the HCV in dialysis units is not a rare event observed world wide. However, the detection of acute HCV infection continues to be a challenge among this population.

The present study of haemodialysis patients lasted for 41 months and showed a high incidence of new hepatitis C cases over a short period, characterizing an outbreak of HCV infection in this unit.

During the investigation (January 2002 to May 2005), 32 new cases of acute hepatitis C were diagnosed. The detection of acute HCV infection was carried out through monthly ALT and anti-HCV markers observation. ALT elevations at the initial stages of HCV infection varied among patients, reaching a maximum of 325 IU/L, preceding up to 4 months the appearance of anti-HCV in the serum. It should be noted that the antibody in hemodialysis patients makes slower appearance than in non-hemodialysis patients and seroconversion may depend on each patient response. Therefore HCV RNA screening would be advisable, but a cost-effective analysis should be considered in this situation. To rule out false-positive ELISA in patients tested negative for HCV RNA, the immunoblot assay is still useful as a supplemental assay [19]. However, this is sometimes not available everywhere due to costs of the method.

The baseline levels of ALT in patients on dialysis are much lower than that of the general population. The study by Guh et al [10], suggests that the cutoff for the detection of HCV infection should be 16 UI/I for ALT and 18UI/I for aspartate transaminase (AST). Gouveia et al [20], suggest that higher normal limits of ALT should be reduced to 60% of conventional limits, when evaluating renal failure patients on dialysis. These lower levels are not yet fully accepted. Our results confirm the importance of monthly surveillance of ALT and that in even mild increases of the monthly standard can indicate acute HCV infection.

The confirmation of acute HCV infection is made by the detection of viremia. In our study confirmation was made by the detection of HCV-RNA using the Polymerase Chain Reaction (PCR). PCR is a good diagnostic test of HCV infection in chronic renal patients, despite observing variations in up to two thirds of patients possibly related to PCR inhibitors, that could be more frequent in patients on dialysis [21]. If positive, it suggests the presence of infection and makes possible the evaluation of the genotype. Another difficulty of this exam is that it is carried out in specialized laboratories prepared for molecular biology studies. Besides the technical characteristics of this method, another problem related to the intermittent viremia in these patients, is the need to carry out the exams repeatedly, representing a technical limitation and increasing costs.

Acute HCV infection cases, with confirmed viremia and without viral clearance up after 12 weeks, should be carefully evaluated for treatment, thus reducing the risks of the patients becoming chronic, which could worsen life expectancy, as in the response to renal transplantation.

Many investigators recommend interferon treatment for 24 weeks [22,23]. Use of Ribavirin is not indicated in these patients because of the risk of hemolytic anemia [24,25]. Pegylated interferon alfa 2b is excreted by the kidneys, therefore we used a lower dose (1 mcg per Kg) [26]. In our study, the selected patients could not be standardized in relation to the onset of the disease. Of the 10 treated patients, four had sustained virological response, three did not responded to treatment, one died during treatment, owing to fistula infection and pneumonia, however without evidence of leukopenia. One patient [27] with high ALT and seroconversion anti-HCV, received treatment, and had all collected samples clear of HCV-RNA. Another patient [25], whose samples of pre-treatment HCV-RNA were collected 3 months prior to treatment, developed non-detectable PCR, however, these cases cannot confirmed if it was due to therapeutic response or natural causes.

In the light of a HCV infection outbreak seen in our haemodialysis unit, we adopted concomitant measures for its identification, as well as the containment of new cases appearing. These measures included the retraining of personnel in relation to universal biosafety guidelines and universal precautions [6] and the importance of the health-care workers and controls in the nosocomial transmission. The patients were isolated in turns with positive serology for anti-HCV, this separation was also done while patients were in transfer because of possible vascular bleeding due to heparinization during dialysis [27]. All equipment utilized on patients was also separated. The procedure for sterilizing the dialysis machines was revised; the reprocessing device was modified using automatic and individual reprocessing in cases of negative serology.

In the haemodialysis units, the risk of infection has been related to the patients and units, characterizing nosocomial transmission [2,28].

Pinto dos Santos et al [3] suggest that HCV transmission in haemodialysis units can be reduced by isolation of HCV positive patients, using separate machines and isolating the reused lines. As well as these investigators we observed the reduction of new cases after the isolation of cases in specific shifts. On the other hand, Jadoul et al [15] reinforce that only the following of universal rules and precautions would be enough to prevent transmission of HCV in haemodialysis units, without the need to isolate infected patients.

The effectiveness of isolation measures to prevent HCV infections is controversial. The strict observance of universal precautions has been sufficient to prevent nosocomial transmission of HCV. However, when selective isolation measures, such as, separation of equipment and patients, in a specific area of the unit, have been adopted, specially in units of high prevalence of HCV and also when the patient/health-care worker ratio is low, this involuntary favors the breach of universal precautions [1,28]. Despite some controversy, in our case, the measures established were fundamental for the containment of the hepatitis C outbreak, the last case being registered 12 months from the end of the study.

## Conclusion

It can be concluded that nosocomial transmission continues to have a major role in the transmission of HCV in haemodialysis units, that acute HCV infection be monitored and measures established to control the outbreak of new cases, and those cases with persistent viremia after 12 months, should be included in treatment procedures.

## Abbreviations

HCV – hepatitis C virus

ALT – alanine aminotransferase

PCR – Polymerase Chain reaction

IFN – interferon

## Competing interests

The author(s) declare that they have no competing interests.

## Authors' contributions

ME and FJC conceived, designed, carried out the entire study in addition to the standardization of the protocol and preparation of the manuscript. SKON, FMM, MMSG, IMVGC, JRRP participated in the design of the study, preparation of the manuscript and performed the statistical analysis. All authors read and approved the final manuscript.

## Pre-publication history

The pre-publication history for this paper can be accessed here:



## References

[B1] Barril G, Traver JA (2003). Decrease in the hepatitis C virus (HCV) prevalence in hemodialysis patients in Spain: effect of time, initiating HCV prevalence studies and adoption of isolation measures. Antiviral Res.

[B2] McLaughlin KJ, Cameron SO, Good T, McCruden E, Ferguson JC, Davidson F, Simmonds P, Mactier RA, McMillan MA (1997). Nosocomial transmission of hepatitis C virus within a British dialysis centre. Nephrol Dial Transplant.

[B3] dos Santos JP, Loureiro A, Cendoroglo Neto M, Pereira BJ (1996). Impact of dialysis room and reuse strategies on the incidence of hepatitis C virus infection in haemodialysis units. Nephrol Dial Transplant.

[B4] Castell J, Gutierrez G (2005). [Outbreak of 18 cases of hepatitis C in a hemodialysis unit]. Gac Sanit.

[B5] Delarocque-Astagneau E, Baffoy N, Thiers V, Simon N, de Valk H, Laperche S, Courouce AM, Astagneau P, Buisson C, Desenclos JC (2002). Outbreak of hepatitis C virus infection in a hemodialysis unit: potential transmission by the hemodialysis machine?. Infect Control Hosp Epidemiol.

[B6] (2001). Recommendations for preventing transmission of infections among chronic hemodialysis patients. MMWR Recomm Rep.

[B7] Fabrizi F, Martin P, Dixit V, Brezina M, Cole MJ, Vinson S, Mousa M, Gitnick G (2000). Biological dynamics of viral load in hemodialysis patients with hepatitis C virus. Am J Kidney Dis.

[B8] Fabrizi F, Bunnapradist S, Lunghi G, Martin P (2003). Kinetics of hepatitis C virus load during hemodialysis: novel perspectives. J Nephrol.

[B9] Galan F, Perez-Gracia MT, Lozano A, Benavides B, Fernandez-Ruiz E, Rodriguez-Iglesias MA (1998). A 3-year follow-up of HCV-RNA viraemia in haemodialysis patients. Nephrol Dial Transplant.

[B10] Guh JY, Lai YH, Yang CY, Chen SC, Chuang WL, Hsu TC, Chen HC, Chang WY, Tsai JH (1995). Impact of decreased serum transaminase levels on the evaluation of viral hepatitis in hemodialysis patients. Nephron.

[B11] Saab S, Martin P, Brezina M, Gitnick G, Yee HF (2001). Serum alanine aminotransferase in hepatitis c screening of patients on hemodialysis. Am J Kidney Dis.

[B12] Yasuda K, Okuda K, Endo N, Ishiwatari Y, Ikeda R, Hayashi H, Yokozeki K, Kobayashi S, Irie Y (1995). Hypoaminotransferasemia in patients undergoing long-term hemodialysis: clinical and biochemical appraisal. Gastroenterology.

[B13] Chomczynski P, Sacchi N (1987). Single-step method of RNA isolation by acid guanidinium thiocyanate-phenol-chloroform extraction. Anal Biochem.

[B14] Campiotto S, Pinho JR, Carrilho FJ, Da Silva LC, Souto FJ, Spinelli V, Pereira LM, Coelho HS, Silva AO, Fonseca JC, Rosa H, Lacet CM, Bernardini AP (2005). Geographic distribution of hepatitis C virus genotypes in Brazil. Braz J Med Biol Res.

[B15] Jadoul M, Cornu C, van Ypersele de Strihou C (1998). Universal precautions prevent hepatitis C virus transmission: a 54 month follow-up of the Belgian Multicenter Study. The Universitaires Cliniques St-Luc (UCL) Collaborative Group. Kidney Int.

[B16] Petrosillo N, Gilli P, Serraino D, Dentico P, Mele A, Ragni P, Puro V, Casalino C, Ippolito G (2001). Prevalence of infected patients and understaffing have a role in hepatitis C virus transmission in dialysis. Am J Kidney Dis.

[B17] Tokars JI, Miller ER, Alter MJ, Arduino MJ (1998). National surveillance of dialysis associated diseases in the United States, 1995. Asaio J.

[B18] Agência Nacional de Vigilância Sanitária - ANVISA (2004). Estabelece o Regulamento Técnico para o Funcionamento dos Serviços de Diálise.

[B19] (2002). National Institutes of Health Consensus Development Conference Statement: Management of hepatitis C: 2002--June 10-12, 2002. Hepatology.

[B20] Gouveia EC, Lopes EP, Moura I, Cruz M, Kosminsky L, Pernambuco JR (2004). [Identification of the cutoff value for serum alanine aminotransferase in hepatitis C screening of patients with chronic renal failure on hemodialysis]. Rev Soc Bras Med Trop.

[B21] Pol S, Vallet-Pichard A, Fontaine H, Lebray P (2002). HCV infection and hemodialysis. Semin Nephrol.

[B22] Gursoy M, Gur G, Arslan H, Ozdemir N, Boyacioglu S (2001). Interferon therapy in haemodialysis patients with acute hepatitis C virus infection and factors that predict response to treatment. J Viral Hepat.

[B23] Urbanek P, Tesar V, Prochazkova-Francisci E, Lachmanova J, Marecek Z, Svobodnik A (2004). Treatment of early diagnosed HCV infection in hemodialyzed patients with interferon-alpha. Treatment of hepatitis C. Blood Purif.

[B24] Espinosa M, Rodriguez M, Martin-Malo A, Alvarez de Lara MA, Gonzalez R, Lopez-Rubio F, de la Mata M, Aljama P (2001). Interferon therapy in hemodialysis patients with chronic hepatitis C virus infection induces a high rate of long-term sustained virological and biochemical response. Clin Nephrol.

[B25] Nomura H, Sou S, Tanimoto H, Nagahama T, Kimura Y, Hayashi J, Ishibashi H, Kashiwagi S (2004). Short-term interferon-alfa therapy for acute hepatitis C: a randomized controlled trial. Hepatology.

[B26] Foster GR (2004). Review article: pegylated interferons: chemical and clinical differences. Aliment Pharmacol Ther.

[B27] Yang CS, Chang HH, Chou CC, Peng SJ (2003). Isolation effectively prevents the transmission of hepatitis C virus in the hemodialysis unit. J Formos Med Assoc.

[B28] Pujol FH, Ponce JG, Lema MG, Capriles F, Devesa M, Sirit F, Salazar M, Vasquez G, Monsalve F, Blitz-Dorfman L (1996). High incidence of hepatitis C virus infection in hemodialysis patients in units with high prevalence. J Clin Microbiol.

